# Integrating Endovascular Drug Delivery into the Therapeutic Landscape of Glioblastoma

**DOI:** 10.3390/cancers18142278

**Published:** 2026-07-15

**Authors:** Zahra Hasanpour-Segherlou, Abdolreza Alikhani, Luca Bertola, Connor Rupp, Maya Haghighi, Jerick Kim, Clayton Rawson, Andrea Baloi, Fatemehsadat Hosseini, Mehrdad Pahlevani, Brandon Lucke-Wold

**Affiliations:** 1 Department of Neurosurgery, University of Florida, Gainesville, FL 32608, USA; 2 Tongji Medical College, Huazhong University of Science and Technology, Wuhan 430030, China; reza.alikhani.med@gmail.com (A.A.); i202420173@hust.edu.cn (F.H.); 3 College of Medicine, University of Florida, Gainesville, FL 32608, USA; lucabertola@ufl.edu (L.B.); crupp@ufl.edu (C.R.);; 4 School of Medicine, Wayne State University, Detroit, MI 48201, USA; ia5435@wayne.edu; 5 College of Medicine, Noorda College of Osteopathic Medicine, Provo, UT 84604, USA; 6 Victor Babes University of Medicine and Pharmacy Timisoara, 300041 Timisoara, Romania; andrea.baloi@student.umft.ro; 7 Department of Neurosurgery, Jamaica Hospital Medical Center, New York City, NY 11418, USA; mpahleva@jhmc.org

**Keywords:** Glioblastoma, endovascular procedures, chemotherapy

## Abstract

Glioblastoma (GBM) is the most common and aggressive primary brain tumor, characterized by poor prognosis and a median survival of 12–18 months despite standard therapies such as surgery, radiation, and temozolomide chemotherapy. Its high cellular heterogeneity, along with complex mechanisms of therapy resistance, presents significant challenges for effective treatment. Conventional systemic chemotherapy is limited by the blood–brain barrier (BBB), systemic toxicity, and insufficient drug penetration into the tumor microenvironment. Emerging therapeutic strategies aim to overcome these barriers through novel chemotherapeutic agents, targeted therapies, immunotherapies, and smart drug delivery systems. Endovascular drug delivery, particularly super-selective intra-arterial cerebral infusion (SSIACI), offers a minimally invasive approach to directly target the tumor vasculature, potentially increasing drug concentration at the tumor site while reducing systemic exposure. Complementary techniques, such as MR-guided focused ultrasound, hyperosmotic disruption, and nanoparticle-based carriers, are being explored to enhance BBB penetration and retention of therapeutics within the tumor. Ongoing clinical trials and translational studies provide insights into optimizing these approaches, with future directions focused on precision medicine, biomarker-driven patient selection, and combination therapies. Integrating endovascular strategies with innovative chemotherapies and immunotherapies may transform GBM management, but further research is required to establish their efficacy and safety in clinical practice.

## Simple Summary

Glioblastoma is the most common and aggressive malignant brain tumor in adults. Despite surgery, radiation therapy, and chemotherapy, most patients experience tumor recurrence, and survival remains limited. One of the greatest challenges in treating glioblastoma is delivering enough medication to the tumor because the brain is protected by a natural barrier that blocks many drugs from reaching their target. This review examines emerging treatment strategies designed to overcome this obstacle. We focus on innovative methods that deliver therapies directly to the blood vessels supplying the tumor, allowing higher drug concentrations to reach cancer cells while potentially reducing side effects in the rest of the body. We also discuss new chemotherapy agents, targeted treatments, immunotherapies, and advanced drug delivery technologies. By summarizing recent advances and ongoing research, this review highlights promising approaches that may improve future treatment options and outcomes for patients with glioblastoma.

## 1. Introduction

Glioblastoma (GBM) is the most common and aggressive malignant primary brain tumor in adults, accounting for approximately 50% of all malignant brain and central nervous system (CNS) tumors in the United States [1,2]. GBM imposes a multidimensional clinical burden due to its rapid progression, high mortality, and profound neurological impact [1,2]. With an age-adjusted incidence of 3.2 per 100,000 individuals, the median survival ranges from 12 to 18 months, even with aggressive medical intervention [1,2,3,4]. The burden extends beyond survival, as 90% of patients experience severe and progressive neurological symptoms that significantly limit patient independence and quality of life, with the most common symptoms being seizures (37%), cognitive deficits (36%), drowsiness (35%), dysphagia (30%), headache (27%), confusion (27%), aphasia (24%), motor deficits (21%), fatigue (20%), and dyspnea (20%) [5,6]. Additionally, GBM accounts for the highest years of life lost among all CNS malignancies, averaging nearly 20 years per patient [7]. The economic burden is also substantial, with costs exceeding $160,000 per patient in the first year of treatment in the United States, alongside high healthcare utilization and frequent hospitalizations [8]. Caregivers are similarly affected, experiencing high rates of psychological distress due to the progressive cognitive and functional decline of their patients [8].

Given the substantial clinical and socioeconomic burden associated with GBM, considerable efforts have focused on developing multimodal therapeutic strategies to improve survival and preserve neurological function. The current standard of care for GBM is the Stupp Protocol, which involves maximal safe surgical resection followed by radiotherapy with concurrent and adjuvant temozolomide chemotherapy [9]. The concurrent phase follows, consisting of daily oral temozolomide at 75 mg/m^2^ for 42 consecutive days [9]. After a 4-week break, the adjuvant phase follows consisting of temozolomide at 150–200 mg/m^2^ for 5 consecutive days every 28 days for 6 cycles [9]. This multimodal approach has remained the cornerstone of GBM management since its introduction and offers modest improvements in survival, increasing the median overall survival to approximately 14.6 months compared with 12.1 months with radiotherapy alone [9]. However, complete surgical eradication is rarely feasible due to the diffuse and invasive nature of the tumor, which extends microscopically beyond radiographically and histological visible margins. Despite aggressive multimodal treatment, GBM is characterized by nearly universal recurrence and the development of therapeutic resistance. Tumor heterogeneity contributes significantly to treatment failure, enabling subpopulations of cells to evade therapies. Median progression-free survival is approximately 7 months, and most recurrences occur locally due to the tumor’s highly infiltrative nature [2]. Resistance is driven by multiple mechanisms, including tumor heterogeneity, glioma stem cells, and molecular factors such as MGMT-mediated DNA repair. Consequently, prognosis remains poor, with median survival of 12–18 months and 5-year survival rates below 10% [3,4].

These persistent limitations have generated increasing interest in treatment strategies that pair more effective pharmacologic agents with improved methods of intracranial drug delivery. Although BBB modulation, intra-arterial infusion, nanomedicine, and emerging chemotherapeutic or biologic agents have each been discussed in the GBM literature, they are often considered as separate therapeutic domains rather than as interdependent components of a delivery-centered treatment strategy. This review addresses that gap by examining how endovascular delivery platforms, particularly super-selective intra-arterial cerebral infusion, may be integrated with novel chemotherapeutics, targeted agents, biologics, and nanoparticle-based systems to improve regional tumor exposure while limiting systemic toxicity. By linking the biological barriers of GBM with catheter-based delivery principles and translational evidence, this review aims to clarify the current rationale, limitations, and future direction of endovascular targeting strategies for GBM.

## 2. Methods

A comprehensive literature search was performed across PubMed, Scopus, and Web of Science databases from inception through May 2026. Studies were included if they addressed one or more of the following topics: (1) the pathophysiology of the GBM tumor microenvironment and mechanisms of therapeutic resistance; (2) limitations of conventional systemic chemotherapy for GBM; (3) emerging chemotherapeutic, targeted, or immunotherapeutic agents under investigation for GBM; (4) strategies for overcoming the blood–brain barrier (BBB), including osmotic disruption, focused ultrasound, nanoparticle-based platforms, and receptor-mediated transcytosis; (5) endovascular and intra-arterial drug delivery techniques; and (6) clinical trials, translational studies, and future directions in personalized medicine for GBM. Eligible study designs included randomized controlled trials, non-randomized clinical studies, preclinical investigations, systematic reviews, meta-analyses, case series, and case reports. Review articles, editorials, and expert commentaries were also considered when they provided relevant mechanistic or contextual insights. No restrictions were placed on language; however, all identified literature was published in English.

## 3. Tumor Microenvironment in GBM

The tumor microenvironment (TME) of GBM is a dynamic ecosystem that is now recognized as a principal driver of therapeutic failure, extending well beyond the intrinsic genetic features of tumor cells. The TME consists of cellular and non-cellular elements, including immune cells, neurons, astrocytes, the extracellular matrix, and the BBB, that collectively shape tumor growth, invasion, and treatment resistance [10]. A thorough understanding of the TME is therefore a prerequisite for designing delivery strategies and pharmacological agents that achieve durable tumor control.

A defining feature of GBM is its profound intratumoral heterogeneity, driven in large part by a subpopulation of GBM stem cells (GSCs) [11]. GSCs possess self-renewal capacity, multipotency, and the ability to continuously regenerate tumor diversity [11]. They also exhibit distinct molecular signatures, enhanced DNA repair, and metabolic adaptations that shield them from conventional treatments [12]. Critically, GSCs do not exist in isolation. They occupy specialized perivascular and hypoxic niches that sustain stemness, promote immunosuppression, and facilitate angiogenesis [12], with Notch, Wnt/β-catenin, and Hedgehog signaling among the key pathways governing their maintenance and plasticity [13]. This self-renewing, therapy-resistant reservoir is widely regarded as the primary source of tumor recurrence [13].

Hypoxia is an inevitable consequence of GBM’s rapid, disorganized growth and represents a major orchestrator of the immunosuppressive TME [14]. It triggers the release of vascular endothelial growth factor (VEGF), which worsens vascular permeability, inflammation, and edema in a feedback loop that simultaneously fuels angiogenesis and promotes stem-like characteristics in glioma cells by activating the Notch pathway in the perivascular niche [10]. The resulting vasculature is structurally abnormal, tortuous, leaky, and heterogeneously distributed, impairing drug delivery by creating regions of elevated interstitial pressure and poor perfusion [15]. Hypoxia further drives GSC self-renewal, chemoresistance, and immune evasion, while metabolic reprogramming toward lactic acid production suppresses antitumor immune responses [15].

GBM is characterized by one of the most immunosuppressive microenvironments among solid tumors. Tumor-associated macrophages, myeloid-derived suppressor cells (MDSCs), and regulatory T cells collectively create an immunosuppressive niche that sustains tumor growth and enables immune evasion [16]. Tumor-associated macrophages, which can constitute up to half the tumor mass, are preferentially polarized toward a pro-tumoral phenotype [16]. In hypoxic regions, they upregulate glycolytic genes and fatty acid oxidation pathways to sustain their survival while releasing immunosuppressive cytokines such as interleukin-10 and TGF-β [17]. MDSCs further suppress cytotoxic T cell function, and GSCs themselves amplify MDSC and macrophage infiltration through epigenetic upregulation of chemokine signaling, reinforcing an immune-privileged tumor niche [18].

The TME drives therapeutic resistance through multiple, reinforcing mechanisms. The extracellular matrix, rich in hyaluronic acid and fibronectin, serves as a physical barrier that limits drug penetration and reduces the efficacy of both small molecules and biologics [10].

These barriers are spatially heterogeneous rather than uniform: hypoxic and necrotic niches are frequently associated with abnormal, compressed, or poorly perfused vessels, which can reduce convective transport and undermine uniform distribution of intra-arterially delivered agents despite selective catheter positioning. In contrast, infiltrative tumor margins may retain a relatively intact BBB, restricting penetration of both systemically administered and regionally delivered therapeutics. Elevated interstitial pressure, overexpression of efflux pumps in BBB endothelial cells, and hypoxia-driven metabolic adaptation further compound these delivery failures by promoting regional underdosing and the persistence of therapy-resistant cellular populations [10,19]. Collectively, these features explain why GBM heterogeneity is not only a mechanism of cellular resistance but also a determinant of uneven drug distribution, and why innovative delivery strategies are essential components of next-generation GBM therapeutic strategies [10].

## 4. Limitations of Conventional Chemotherapy

Temozolomide and bevacizumab are among the only chemical therapeutics approved for primary and recurrent GBM [20]. Temozolomide is an alkylating agent that covalently adds methyl groups to purines, thereby fragmenting DNA and inducing cell death [20]. Anti-angiogenic therapies targeting the VEGF signaling pathway have been extensively investigated as a strategy to inhibit tumor vascularization and growth [21]. Bevacizumab is a monoclonal antibody that binds to and inhibits VEGF, the primary growth factor for tumor angiogenesis [22]. Temozolomide’s baseline efficacy is attributable to its pharmacodynamics [23]. It can cross the BBB through passive diffusion. This is due to its low molecular weight and lipophilic properties; concentrations of Temozolomide in the CNS are 30% of serum concentrations, and its active metabolite MITC does not penetrate the CNS at all [23]. While 100% orally bioavailable, it has a half-life of only 1.8 h, necessitating frequent dosing. Temozolomide is well tolerated; however thrombocytopenia and lymphopenia are commonly its dose-limiting toxicity, with about 4% of cycles requiring a dose reduction and 28% of cycles requiring treatment delay [23].

Bevacizumab is similarly well-tolerated, and its dose-limiting toxicities are usually hypertension and proteinuria, with rare adverse effects such as thromboembolic events and gastrointestinal perforation [22]. Pharmacokinetically, bevacizumab has substantially longer systemic exposure than temozolomide, with an estimated half-life of approximately 20 days, compared with temozolomide’s 1.8-h half-life [22,23].

Despite transient clinical and radiological improvements, anti-angiogenic therapies such as bevacizumab have failed to demonstrate a significant improvement in overall survival in GBM [21,24]. The drawback of bevacizumab stems from its mechanism of action rather than its pharmacodynamics. Its use necessarily selects for tumor cells resistant to its antiangiogenic effects and can result in more aggressive phenotypes that evade the anti-VEGF effects via GSCs, creating pseudovascular channels to further tumor growth [22]. Beyond the intrinsic pharmacologic limitations of these agents, multiple structural and molecular barriers within GBM further restrict therapeutic efficacy. Physical barriers like the BBB and the blood–tumor barrier (BTB) exist alongside molecular barriers such as transmembrane efflux proteins, genetic and epigenetic modifications.

The BBB is a sophisticated interface that maintains neural homeostasis and comprises specialized endothelial cells, pericytes, and astrocyte end-feet. The integrity of the BBB is altered but not destroyed in GBM [25]. As the tumor grows, the core disrupts the BBB, but the invasive edges remain largely protected from therapeutics by an intact BBB. Furthermore, the solid-tumor nature increases hydrostatic pressure and is further exacerbated by vascular leakiness and glymphatic obstruction [25]. This pressure gradient forces fluid away and any large molecules like monoclonal antibodies, which rely on convection rather than passive diffusion, along with it [25]. Molecular barriers to chemotherapy include efflux pumps and pathologic cellular signaling. Efflux pumps, such as P-glycoprotein (P-gp), breast cancer resistance protein, and multidrug resistance protein 1, reside in the BBB and actively pump a wide variety of lipophilic chemotherapeutics [26]. Further, P-gp activity is observed at both the BTB and BBB, counteracting the vulnerability associated with more permeable vasculature [27]. MGMT demethylates DNA, thereby protecting against the molecular lesions induced by Temozolomide [27]. Promoter methylation decreases expression and increases susceptibility to Temozolomide [27]. Recurrent GBMs show increased expression of MGMT whether through promoter demethylation or genomic rearrangement [27]. The mismatch repair signaling pathway is also of particular importance because, when unable to repair temozolomide-induced DNA damage, it induces apoptosis. Derangement of the mismatch repair signaling pathway is linked to hypermutation [28]. Lastly, the heterogeneity of the tumor microenvironment is a major challenge. GSCs are a subset of GBM cells that exhibit cellular plasticity and unlimited proliferative potential. They contribute to treatment resistance by creating functionally distinct subpopulations within the tumor that may include combinations of the previously mentioned mechanisms [25].

## 5. Emerging Chemotherapeutic and Targeted Agents

To address the limitation of temozolomide, novel alkylating agents have been developed to enhance therapeutic efficacy in GBM [29]. One example is dianhydrogalactitol, a promising alkylating agent that exerts its cytotoxic effects through MGMT-independent DNA crosslinking [29]. Despite promising preclinical data, clinical benefits remain modest, warranting further investigation [30].

Targeted therapeutic strategies are designed to selectively inhibit dysregulated signaling pathways driving GBM progression [31]. The Epidermal Growth Factor Receptor (EGFR) signaling pathway is frequently altered in GBM and plays a critical role in promoting cellular proliferation and survival [31]. Despite a strong biological rationale, EGFR-targeted therapies have shown limited clinical efficacy in GBM, largely due to intrinsic tumor heterogeneity and adaptive resistance mechanisms [31].

Immune checkpoint inhibitors such as nivolumab (PD-1 blockade) have been evaluated in GBM to enhance anti-tumor immune responses; however, their clinical efficacy has remained limited [24]. Despite promising preclinical findings, CAR-T cell therapy targeting tumor-associated antigens such as EGFR variant III has been investigated in GBM; however, this approach faces significant challenges, including limited tumor infiltration and intratumoral antigen heterogeneity [32].

Antibody–drug conjugates are targeted therapeutic agents designed to enhance tumor-specific cytotoxicity while minimizing systemic toxicity by linking monoclonal antibodies to cytotoxic payloads, enabling selective delivery of chemotherapy to tumor cells [33]. Antibody–drug conjugates are being refined for CNS application, with emerging proteomic and transcriptomic profiling identifying clinically actionable targets in gliomas—including HER2, TROP2, FOLR1, CLDN6, HER3, and B7-H3—that are not detected by DNA-based next-generation sequencing alone [34]. In GBM, antibody–drug conjugates have been investigated as a strategy to improve drug delivery across the BBB; however, their clinical translation remains limited by restricted tumor penetration and intratumoral heterogeneity [33]. Despite promising targeting capability, antibody–drug conjugates in GBM remain limited by delivery barriers and tumor heterogeneity, highlighting the need for further optimization to overcome these biological and delivery-related challenges [33]. Nanomedicine-based drug delivery systems have emerged as a promising strategy to overcome biological barriers, including the BBB, and to improve therapeutic delivery in GBM [35]. Stimuli-responsive nanocarriers have been developed to enable controlled and site-specific drug release triggered by tumor microenvironmental conditions, thereby improving therapeutic precision and efficacy in GBM [35]. Despite promising preclinical results, nanomedicine-based approaches require further clinical validation before widespread application in GBM [35].

Molecularly defined subsets of GBM harboring actionable alterations—including BRAF V600E mutations (1–2% of cases), NTRK gene fusions (1–2%), and FGFR alterations—can be treated with tumor-agnostic targeted agents such as dabrafenib/trametinib, entrectinib, larotrectinib, repotrectinib, and erdafitinib, which have demonstrated radiographic responses in case reports and non-randomized studies [36,37]. Furthermore, adaptive platform trial frameworks such as GBM AGILE and INSIGhT are accelerating the evaluation of multiple novel agents within molecularly stratified cohorts, enabling efficient identification of effective therapies while reducing the time and cost of traditional sequential trial designs [36]. Collectively, these advances underscore a translational shift from empirical cytotoxic monotherapy toward multimodal, biomarker-informed regimens that integrate targeted agents, next-generation immunotherapies, and precision-guided endovascular delivery strategies to address the complex biology and therapeutic resistance of GBM.

## 6. Strategies to Overcome BBB

BBB constitutes a critical physiological barrier that significantly limits the delivery of systemically administered therapeutic agents to brain tumors [38]. Intra-arterial (IA) administration of hyperosmolar mannitol transiently increases BBB permeability through osmotic shrinkage of endothelial cells, resulting in reversible disruption of tight junctions [39]. This technique has been clinically utilized to improve the penetration of chemotherapeutic agents in patients with malignant brain tumors [39]. Despite its clinical utility, mannitol-induced BBB disruption is nonspecific and transient and has been associated with significant neurological adverse events, including seizures and neurotoxicity, thereby underscoring the need for more targeted delivery strategies [39].

Magnetic resonance-guided focused ultrasound, in combination with intravenously administered microbubbles, represents a non-invasive, image-guided, spatially targeted physical approach to reversibly disrupt the BBB [40]. Oscillation of intravenously administered microbubbles within the vascular lumen generates localized shear stress on endothelial cells, leading to transient opening of tight junctions and reversible modulation of the BBB under real-time MRI guidance [41].

At the preclinical level, focused ultrasound has been shown to significantly enhance the delivery of therapeutic agents across the BBB, increasing brain drug uptake by several orders of magnitude without causing significant tissue damage [40]. Despite its therapeutic potential, this technique is limited by high equipment costs, the requirement for specialized technical expertise, and restricted availability in clinical settings, particularly outside major academic centers [40].

Microbubble-enhanced transcranial focused ultrasound (MB-FUS) activates systemically administered microbubble resonators to generate mechanical stress on capillary endothelium, enabling controlled BBB opening and enhanced drug penetration into peritumoral infiltrative zones [42]. The BT008NA phase 1/2 multicenter trial demonstrated that MB-FUS combined with adjuvant temozolomide was associated with significantly improved overall survival and progression-free survival compared to a matched external control group receiving temozolomide alone, representing the first comparative report to suggest a survival benefit from adding FUS-mediated BBB disruption to chemotherapy in newly diagnosed high-grade glioma [42]. Additionally, implantable ultrasound devices have been investigated for repeated BBB opening to facilitate delivery of otherwise brain-impenetrant agents such as albumin-bound paclitaxel, which is approximately 1400 times more potent than temozolomide in vitro but does not cross the intact BBB [43]. Perfusion-guided endovascular super-selective intra-arterial infusion (PG-ESIA), which co-registers MRI with cone-beam CT for optimal vessel selection and targeted delivery confirmation, has been developed to further enhance the precision of intra-arterial therapeutic delivery [44]. Beyond conventional chemotherapeutics, the endovascular platform is being expanded to include novel therapeutic payloads such as yttrium-90 radioembolization, oncolytic viruses (e.g., mesenchymal stem cell-loaded Delta-24), and cellular immunotherapies delivered via intra-arterial routes [45,46].

Nanoparticle-based platforms offer a versatile strategy for bypassing the BBB by harnessing endogenous transport mechanisms [47]. Various nanoparticle formulations, including liposomes, polymeric nanoparticles, and inorganic nanoparticles, have demonstrated the ability to traverse the BBB and deliver therapeutic agents to brain tumors [48]. The “Trojan horse” strategy exploits biomimicry by engineering nanoparticles to mimic endogenous proteins or cellular components, thereby enabling them to evade immune surveillance and facilitate transport across the BBB [49]. Surface functionalization of nanoparticles with targeting ligands, such as transferrin or cell-penetrating peptides, enhances their ability to traverse the BBB and improves brain targeting efficiency [47]. Despite their therapeutic potential, significant challenges remain, including manufacturing complexity, batch-to-batch variability, and limited clinical scalability, which hinder their widespread clinical translation [48].

Beyond physical and nanoparticle-assisted BBB modulation strategies, biologically mediated transport systems have also emerged as promising approaches for enhancing selective therapeutic delivery to the brain. Receptor-mediated transcytosis represents a targeted transport mechanism that exploits highly expressed receptors on brain endothelial cells to facilitate the selective and efficient delivery of therapeutic agents across the BBB [50]. The most extensively studied receptor targets for this approach include the transferrin receptor, insulin receptor, and low-density lipoprotein receptor-related protein-1 [51]. Conjugation of therapeutic agents to receptor-specific ligands enables selective brain delivery, facilitating receptor-mediated transcytosis, thereby preserving the integrity of the BBB [50]. This strategy has gained increasing attention for enabling the transport of large therapeutic biologics, such as monoclonal antibodies and enzymes, across the BBB while preserving its functional integrity [50,51] (Table 1).

## 7. Rationale for Endovascular Drug Delivery

Given the substantial biologic and pharmacokinetic barriers limiting systemic therapies, endovascular IA drug delivery has emerged as a promising strategy to enhance regional drug delivery and improve intratumoral drug concentrations in GBM. IA drug delivery is rationalized by three fundamental principles: avoidance of first pass metabolism through IA drug delivery, avoidance of systemic drug dilution and metabolism, and precise tumor targeting through modern catheter technology. IA drug delivery allows direct injection of chemotherapeutic agents into tumor-supplying arteries, rapidly increasing intratumoral concentration while maintaining decreased systemic drug exposure [62]. Direct injection of the agent into the tumor capillary bed enables the drug to achieve high regional tissue concentrations at significantly lower doses than would be required with systemic administration [63]. The pharmacokinetic advantage of IA delivery is primarily driven by first-pass drug uptake, in which the agent encounters the tumor vasculature before entering systemic circulation. This maximizes the concentration gradient across the capillary bed during initial delivery [63,64]. IA administration of melphalan showed a significant first-pass advantage over intravenous delivery, with a short plasma half-life, making it well-suited for IA administration [65]. Chemotherapeutic agents that are rapidly metabolized or inactivated are therefore ideal candidates for IA delivery. These pharmacokinetic advantages have provided the foundation for continued technological refinement of selective IA delivery systems and image-guided catheter-based approaches. The method of IA delivery has undergone significant advancements in recent years. Early IA approaches used non-selective intracarotid injection, exposing patients to significant toxicities, including irreversible encephalopathy, visual loss, and white matter necrosis [66]. Modern microcatheter technology now enables safe distal intracranial access and selective catheterization of tumor-feeding arteries with greater precision [67,68]. Perfusion-guided endovascular superselective IA delivery allows real-time confirmation of drug distribution through volumetric perfusion imaging, further enhancing targeting accuracy [44]. Additionally, MRI-guided IA administration has been shown to improve the predictability of drug delivery compared with X-ray guidance alone [69].

## 8. Interventional Oncology Approaches

Interventional oncology approaches for GBM have increasingly focused on overcoming the limitations of systemic chemotherapy by enabling localized drug delivery directly to the tumor vasculature. Among these, super-selective intra-arterial cerebral infusion (SSIACI) has emerged as a promising strategy to enhance regional drug concentration while reducing systemic toxicity [68]. SSIACI delivery is an endovascular approach that enables administration of chemotherapeutic agents into tumor-feeding cerebral arteries [68,70]. Despite these advantages, SSIACI delivery remains limited by heterogeneous tumor vascularization, which may result in uneven intratumoral drug distribution in GBM [68]. SSIACI has been investigated with chemotherapeutic agents such as carboplatin for the treatment of recurrent malignant gliomas [70]. Early studies of IA chemotherapy have demonstrated the feasibility of delivering pharmacologic agents directly into cerebral circulation using endovascular techniques [71]. In some protocols, SSIACI is combined with osmotic BBB disruption using mannitol to further enhance chemotherapeutic penetration and increase intratumoral drug concentration [72,73]. Successful implementation of SSIACI depends not only on drug selection but also on meticulous procedural planning, imaging guidance, and careful management of procedure-related complications. The procedure typically begins with digital subtraction angiography (DSA) to identify tumor-feeding cerebral arteries and facilitate endovascular procedural planning [74,75]. A microcatheter is then advanced into tumor-specific arterial feeders, often branches of the internal carotid or vertebrobasilar systems, depending on tumor location [76]. Super-selective positioning is confirmed using DSA, which allows visualization of tumor blush and vascular territories. Once optimal catheter placement is achieved, therapeutic agents, such as chemotherapeutics (e.g., carboplatin, bevacizumab) or emerging biologics, are infused in a controlled manner. Careful hemodynamic monitoring is essential throughout the infusion to prevent reflux and ensure uniform distribution within the tumor bed. Chemotherapeutic agents are then slowly infused through the microcatheter to optimize intratumoral penetration while minimizing reflux into the normal cerebral circulation [74,75]. The selection of chemotherapeutic agent, infusion rate, and catheter positioning is individualized based on tumor vascular anatomy and patient-specific characteristics [74,75].

DSA remains the gold standard imaging modality for guiding IA neurointerventional procedures and ensuring accurate catheter positioning [6]. Safety considerations include prevention of vasospasm, thromboembolism, arterial injury, and non-target embolization [72,77]. As procedural context, prospective cerebral angiography data have reported neurologic complications in 1.3% of 2899 procedures, including transient deficits in 0.7%, reversible deficits in 0.2%, and permanent deficits in 0.5%; however, these rates should be interpreted as angiography-related benchmarks rather than SSIACI-specific toxicity estimates [74].

Neurological complications may involve transient ischemic attacks, focal neurological deficits arterial dissection, vasospasm, and intracranial hemorrhage [72,77,78]. Advanced imaging modalities further enhance the precision of SSIACI by improving procedural planning, intra-procedural guidance, and post-treatment assessment. Pre-procedural MRI and perfusion imaging are used to define tumor vascularity, assess BTB integrity, and guide target vessel selection [79]. Cone-beam CT and IA contrast-enhanced imaging can further improve spatial resolution and confirm adequate perfusion of the tumor territory [80]. Post-procedural imaging, including contrast-enhanced MRI and diffusion-weighted imaging, is essential for evaluating treatment response and detecting early complications such as ischemia or hemorrhage. Functional imaging modalities, including PET, may also be used.

The use of hyperosmotic agents for BBB disruption also carries risks of seizures, transient neurological dysfunction, and endothelial injury [81,82]. To mitigate these risks, meticulous techniques and careful patient selection are critical. Real-time imaging guidance, slow infusion rates, and avoidance of reflux are essential procedural safeguards. Additionally, multidisciplinary collaboration between neurointerventionalists, neurosurgeons, and neuro-oncologists is necessary to optimize outcomes and ensure appropriate integration with systemic and radiation therapies [83]. Despite these challenges, early clinical studies demonstrate that SSIACI is feasible and may improve drug delivery efficiency in GBM. Ongoing trials are focused on refining delivery techniques, identifying optimal therapeutic agents, and integrating SSIACI into multimodal treatment paradigms [82,83].

## 9. Clinical Trials and Translational Evidence

Early-phase clinical trials have primarily focused on evaluating the safety and feasibility of IA drug delivery combined with BBB disruption strategies. In this context, a Phase I trial used SSIACI combined with osmotic BBB disruption in pediatric patients with refractory high-grade gliomas, including diffuse intrinsic pontine glioma and GBM to assess the safety of targeted IA drug delivery across the BBB [84]. IA mannitol (20%) was used to transiently disrupt the BBB, followed by targeted delivery of bevacizumab (15 mg/kg) and cetuximab (200 mg/m^2^) to inhibit VEGF and EGFR pathways, respectively [84]. The treatment was well tolerated in all 13 patients, supporting its feasibility, although efficacy requires further validation. In a large single-center clinical cohort of 70 patients undergoing 139 SSIACI procedures, IA bevacizumab or cetuximab following BBB disruption was shown to be technically feasible and safe in both newly diagnosed and recurrent GBM [85].

Beyond feasibility studies, several ongoing and completed clinical investigations have explored the therapeutic potential of IA rechallenge strategies and repeated SSIACI administration in recurrent GBM. The first reported case of rechallenging recurrent GBM with SSIACI of bevacizumab following prior treatment failure demonstrated potential therapeutic response [86]. A 43-year-old patient initially received SSIACI bevacizumab in a Phase I/II trial (NCT01811498) alongside standard chemoradiation but progressed and was later retreated in a separate trial (NCT01269853), receiving SSIACI bevacizumab followed by intravenous therapy. This approach produced a radiographic response, suggesting potential benefits of IA rechallenge strategies and highlighting the need for comparative studies between SSIACI and intravenous delivery. Further investigation into anti-angiogenic resistance mechanisms is also necessary [86]. IA delivery provides higher local drug concentrations at the tumor site, reduces systemic toxicity, and demonstrates an acceptable safety profile when performed in experienced centers [79].

However, it is essential to critically acknowledge that despite decades of investigation, intra-arterial chemotherapy for GBM has consistently failed to demonstrate a definitive survival advantage over intravenous administration. The landmark phase III randomized trial by Shapiro et al. (1992) comparing intra-arterial versus intravenous BCNU in 448 patients with newly diagnosed malignant glioma demonstrated not only no survival benefit but significantly reduced survival in the intra-arterial group (*p* = 0.03), accompanied by irreversible encephalopathy in 9.5% and ipsilateral visual loss in 15.5% of patients [87]. A systematic review and meta-analysis by Chen et al. (2013) of four randomized controlled trials encompassing 460 patients confirmed that intra-arterial chemotherapy was not superior to intravenous chemotherapy in terms of disease control rate, efficacy rate, or 1-, 2-, and 3-year overall survival [88]. More recently, the largest meta-analysis to date by Rahmanipour et al. (2026), synthesizing 155 studies across 1302 records, reported a pooled hazard ratio of 1.07 (95% CI 0.95–1.21) for glioma, indicating no significant survival difference, with extreme between-study heterogeneity and evidence of publication bias (Egger’s *p* = 0.001); the authors concluded that the pharmacologic advantages of intra-arterial delivery have not translated into a consistent survival benefit for gliomas [53]. Similarly, a review of comparative studies by Theodotou et al. (2014) found that intravenous chemotherapy actually produced a statistically higher median overall survival (16.3 months) compared with intra-arterial treatment (14.02 months) in newly diagnosed GBM [89]. These findings collectively underscore that the theoretical pharmacokinetic advantages of intra-arterial delivery—higher local drug concentrations with reduced systemic exposure—have not been borne out in clinical survival endpoints. The reasons underlying these repeated failures are multifactorial and warrant explicit discussion. First, early intra-arterial approaches utilized highly neurotoxic agents such as BCNU via non-selective carotid infusion, resulting in significant white matter necrosis and leukoencephalopathy that offset any potential therapeutic benefit [87]. Second, hemodynamic factors—including drug streaming, rapid first-pass transit, and limited drug-tumor residence time—have been consistently underappreciated, with very few studies incorporating pharmacokinetic modeling or quantitative assessment of actual intratumoral drug delivery [63,68]. Third, the inherent biological complexity of GBM, including intratumoral heterogeneity, diffuse infiltrative growth beyond the vascular territory of any single feeding artery, and adaptive resistance mechanisms, fundamentally limits the efficacy of any regionally targeted delivery strategy [68,79]. Fourth, the scarcity of phase III randomized controlled trials remains a critical limitation; a bibliometric analysis by Rechberger et al. (2021) found that of 20 registered clinical trials of intra-arterial therapy for brain tumors, 45% were phase I, and only a single trial (5%) had reported results [90]. While modern SSIACI with less neurotoxic agents such as bevacizumab and carboplatin, combined with BBB disruption, has demonstrated procedural safety—with a 0.9% symptomatic complication rate across 2991 procedures in one large cohort—and encouraging radiographic response rates, these remain single-arm, non-randomized studies with inherent selection bias, as patients eligible for intra-arterial treatment tend to have more favorable prognostic profiles than ineligible patients [52,54,91,92]. Accordingly, while the evolution from non-selective carotid infusion to modern super-selective microcatheter-based delivery represents a meaningful technical advancement, the clinical evidence base remains insufficient to support claims of therapeutic superiority, and adequately powered, randomized phase III trials comparing SSIACI-based regimens to standard systemic therapy are urgently needed to determine whether these technical refinements translate into genuine survival benefits [53,68,79].

On the other hand, several critical translational barriers impede their clinical implementation and must be explicitly acknowledged. From a manufacturing and cost perspective, advanced therapies such as CAR-T cells require personalized, labor-intensive production processes—including patient-specific leukapheresis, genetic modification, and ex vivo expansion—with vein-to-vein times of approximately 13 to 54 days and costs exceeding $400,000 per patient, creating significant disparities in access based on socioeconomic status and geographic location [93,94]. Manufacturing failures and disease progression during wait times result in 0% to 31% of patients never receiving the intended therapy [94]. Similarly, nanomedicine-based drug delivery systems face challenges of exosome manufacturing scalability, conjugate stability, and immunogenicity that must be resolved before clinical translation [95]. Clinical trial failures are pervasive: a comprehensive survey of 3038 neuro-oncology trials found that 65% were single-group, 51% were nonrandomized, 38% of completed trials failed to meet enrollment targets, and efficacy signals were detected in only 15–23% of completed trials reporting survival outcomes [96]. An analysis of 886 GBM-related trials (2003–2020) found that 19.8% were terminated prior to completion, with participant accrual difficulties accounting for 36% of terminations [97]. Regulatory barriers further complicate the landscape: the 2021 WHO Classification of CNS tumors has yet to be universally adopted across clinical trials, leading to inconsistent patient populations; the required genetic testing panels are unavailable as routine diagnostics in many countries; and the absence of validated biomarkers for patient-specific sensitivity to cell and gene therapies limits both regulatory approval pathways and the ability to identify responding patients early during treatment [98]. Collectively, these interrelated challenges of manufacturing complexity, prohibitive costs, restrictive eligibility criteria, high trial failure rates, and fragmented regulatory frameworks represent formidable obstacles that must be systematically addressed through streamlined manufacturing platforms (e.g., point-of-care and allogeneic “off-the-shelf” products), adaptive trial designs such as GBM AGILE and INSIGhT, international regulatory harmonization, and biomarker-driven patient stratification strategies to accelerate the translation of novel GBM therapeutics from bench to bedside [99,100,101].

Beyond the technical and pharmacokinetic challenges of endovascular drug delivery, several fundamental biological limitations of GBM critically undermine the therapeutic rationale of intra-arterial approaches and warrant explicit discussion. First, intratumoral heterogeneity—encompassing spatial, genetic, and transcriptomic diversity—means that GBM harbors coexisting cell populations with divergent drug sensitivities, such that even high regional drug concentrations achieved via SSIACI are unlikely to eradicate all clonal subpopulations within a given vascular territory [102,103]. Second, the variable and aberrant vascular supply of GBM, characterized by disorganized vessel networks, heterogeneous blood–tumor barrier permeability across different tumor regions, and alternative neovascular mechanisms including vessel co-option, vasculogenic mimicry, and glioma stem cell transdifferentiation into vascular-like structures, fundamentally limits the assumption that catheter-directed delivery can achieve uniform drug distribution throughout the tumor mass [104,105,106]. Third, GBM is defined by its diffusely infiltrative growth pattern, with tumor cells extending well beyond the contrast-enhancing core into surrounding brain parenchyma where the BBB remains essentially intact; these infiltrative margins—which represent the primary site of recurrence—are supplied by normal cerebral vasculature rather than tumor-feeding arteries and are therefore inherently inaccessible to endovascular targeting strategies [68,107]. Fourth, adaptive resistance mechanisms further diminish the efficacy of any delivery approach: chemoradiation induces transition to a vessel co-opting, therapy-resistant cell state (VC-Resist) that homes to perivascular niches and exhibits enhanced DNA damage tolerance through FGFR1-YAP1-dependent pathways; glioma stem cells residing in hypoxic niches upregulate ABC efflux transporters (P-glycoprotein, BCRP) that actively pump chemotherapeutic agents out of tumor cells regardless of intracellular drug concentrations achieved; and therapy-induced mesenchymal transitions and metabolic reprogramming enable surviving tumor cells to evade cytotoxic stress and repopulate the tumor mass [102,108,109,110]. Collectively, these interrelated biological barriers—intratumoral heterogeneity, aberrant and variable vascularity, diffuse infiltration beyond targetable vascular territories, and multifaceted adaptive resistance—represent fundamental constraints that cannot be overcome by technical refinements in catheter positioning or BBB disruption alone and must be explicitly acknowledged when evaluating the therapeutic potential of endovascular approaches for GBM (Table 2).

**Table 2:** Comparative Summary of Chemotherapeutic and Targeted Agents for Glioblastoma: Clinical Outcomes and Evidence.

BBB Disruption Method	Mechanism of Action	Drug Delivery Enhancement	Clinical Efficacy (Survival)	Key Safety Concerns	Evidence Level	Current Limitations	References
IA Hyperosmolar Mannitol + SSIACI	Osmotic shrinkage of endothelial cells → transient tight junction opening	Up to 4× higher tumor drug levels vs. IV	mOS 9.1–25 mo (single-arm); meta-analysis: pooled HR 1.07 (95% CI 0.95–1.21) vs. systemic therapy (no significant survival benefit)	Seizures (37.7% of procedures); stroke (0.5–1.3%); procedure-related complications 15.4%	Phase I/II; systematic reviews and meta-analyses; no Phase III RCTs completed	Nonspecific BBB opening; no proven survival benefit over IV; extreme between-study heterogeneity; requires general anesthesia and specialized centers	[52,53,54,55]
Microbubble-Enhanced Focused Ultrasound (MB-FUS)	Acoustic activation of IV microbubbles → localized shear stress on endothelial cells → reversible tight junction opening	2–5.93-fold increase in brain drug concentration	PFS 2.5–4.11 mo; mOS 10–14 mo (recurrent); BT008NA trial: significantly improved OS and PFS vs. matched external controls (newly diagnosed)	Mild/transient: petechiae, edema; no severe AEs reported	Phase 0–Phase 1/2; one comparative trial (non-randomized external controls)	No RCT-confirmed survival benefit; high equipment costs; requires specialized expertise; limited availability outside academic centers; standardization of parameters needed	[42,55,56]
Implantable Ultrasound Device (SonoCloud)	Pulsed ultrasound from implanted transducer → BBB disruption before IV chemotherapy	BBB disruption confirmed on MRI in 90% of emitters	mPFS 3.1 mo; mOS 14.0 mo from surgery; 1-year OS 58% (recurrent GBM with carboplatin)	Grade 3: pre-syncope, fatigue, wound infection, pain at device connection; no DLTs	Phase 1/2 (single arm)	Requires surgical implantation; single-arm data only; limited to recurrent setting; small sample sizes (*n* = 33)	[57]
Laser Interstitial Thermal Therapy (LITT)	MR-guided thermal ablation → BBB disruption in peritumoral zone via tight junction disruption and upregulated transcytosis	Enhanced BBB permeability for 7–21 days post-procedure; increased caveolae-mediated transcytosis	Primarily used as cytoreductive tool; survival data as BBB-disruption platform still emerging	Procedure-related edema; heat sink effect near vasculature; hemorrhage risk	Preclinical + early clinical (as BBB disruption platform)	Primarily ablative; BBB disruption is secondary effect; limited spatial coverage; heat sink effect limits efficacy near vessels; no RCTs as drug delivery platform	[58,59,60]
Nanoparticle-Based Platforms	Surface-functionalized carriers (liposomes, polymeric NPs) exploit receptor-mediated transcytosis or biomimicry to cross intact BBB	Variable; preclinical: 9-fold increase with FUS combination; 13–17-fold with dual-targeting ligands	No clinical survival data as standalone BBB-crossing strategy for GBM	Immunogenicity; off-target accumulation; hepatotoxicity (transient)	Predominantly preclinical; early Phase I	Manufacturing complexity; batch-to-batch variability; limited clinical scalability; most data preclinical only; regulatory pathway unclear	[61,111,112]
Receptor-Mediated Transcytosis	Conjugation of therapeutics to ligands (transferrin, insulin receptor antibodies) targeting endothelial receptors → selective transport across intact BBB	Preclinical evidence of enhanced brain delivery of large biologics (antibodies, enzymes)	No clinical survival data specific to GBM	Receptor saturation; potential disruption of normal receptor function	Preclinical	Limited clinical translation; receptor saturation at therapeutic doses; competition with endogenous ligands; insufficient clinical data	[113]

## 10. Personalized Medicine and Future Directions

Personalized approaches in GBM are increasingly focused on optimizing patient selection and improving therapeutic delivery through selective IA strategies combined with BBB disruption. In parallel, personalized medicine is increasingly shaped by genomic and transcriptomic profiling to address the profound heterogeneity of GBM. The marked molecular and genetic heterogeneity of GBM remains a major barrier to uniform treatment efficacy, limiting the success of standardized therapeutic approaches [79]. Individualized treatment strategies based on molecular profiling are therefore essential for improving outcomes, as GBM exhibits extensive biological variability. Such profiling may enhance patient stratification for IA-based therapies and support multimodal treatment approaches tailored to tumor-specific characteristics. Additionally, advances in imaging integration, such as real-time perfusion imaging during angiography, and emerging drug delivery platforms, such as nanoparticles, may further improve precision and therapeutic targeting [79].

While the integration of precision oncology, artificial intelligence, and advanced imaging into GBM management holds conceptual promise, the current clinical evidence supporting these approaches remains limited and must be transparently acknowledged. Regarding molecular stratification, systematic screening for actionable molecular alterations in GBM has yielded disappointingly low rates of targeted treatment eligibility (<10% of patients), with clinical efficacy observed in only approximately one-third of those treated and currently limited to BRAF-, VEGFR-, and mTOR-directed therapies; notably, EGFR-targeted approaches have consistently failed despite EGFR being the most frequently altered receptor tyrosine kinase in GBM, owing to poor CNS drug penetration, intratumoral heterogeneity, and pathway redundancies [114,115]. Artificial intelligence applications in GBM have demonstrated promising performance in controlled settings—a meta-analysis of 22 controlled studies (9314 patients) reported that AI-based survival prediction models achieved a pooled C-index of 0.81 compared to 0.69 for conventional prognostic tools (*p* < 0.001), with multimodal AI models integrating imaging, genomic, and clinical data reaching a C-index of 0.87—yet external validation cohorts showed significantly reduced accuracy (C-index 0.75), and only 64% of studies reported acceptable calibration, highlighting critical limitations in generalizability and clinical readiness [116]. The first prospective biopsy-controlled validation of an AI model for mapping GBM infiltration (SupraGlio trial) demonstrated 0.81 accuracy and 0.84 AUC for predicting histologically confirmed tumor infiltration, with postoperative high-risk volume >1.6 cm^3^ predicting shorter overall and progression-free survival; however, this remains a single-center study requiring multicenter validation before clinical implementation [117]. Key implementation barriers include challenges related to data harmonization across institutions, model interpretability for clinical decision-making, computational demands that limit deployment in resource-constrained settings, and the absence of prospective randomized trials demonstrating that AI-guided or molecular profiling–guided treatment decisions improve survival outcomes compared to standard approaches [118,119,120]. Adaptive trial frameworks such as GBM AGILE and INSIGhT represent important methodological advances for accelerating biomarker-stratified drug evaluation, but these platforms are still maturing and have yet to produce practice-changing results for the majority of GBM patients [36]. Collectively, while these technologies represent the most promising frontier for advancing GBM therapy, their current clinical evidence base remains preliminary, and their translation into routine practice will require rigorous prospective validation, standardized implementation protocols, regulatory frameworks for AI-based clinical decision support, and demonstration of meaningful survival benefits in randomized controlled trials [103,118,121].

## 11. Conclusions

Glioblastoma remains one of the most therapeutically challenging malignancies due to its marked heterogeneity, invasive behavior, and the protective effects of the BBB. Although current standard therapies provide modest survival benefits, emerging approaches including targeted therapies, immunotherapy, nanomedicine, and endovascular drug delivery strategies offer promising opportunities to improve treatment efficacy. Among these, SSIACI and BBB disruption techniques have demonstrated the potential to enhance local drug delivery while minimizing systemic toxicity. Continued advances in imaging guidance, molecular profiling, and precision medicine may further optimize patient selection and therapeutic outcomes. However, larger prospective clinical trials and standardized treatment protocols are still required before these strategies can be fully integrated into routine clinical practice.

The convergence of endovascular delivery platforms with complementary BBB-modulation techniques represents a particularly compelling avenue for future investigation. MR-guided focused ultrasound combined with microbubbles has shown the capacity to achieve spatially targeted, reversible BBB opening under real-time imaging guidance, while nanoparticle-based carriers functionalized with receptor-specific ligands can exploit endogenous transcytosis pathways to enhance drug retention within the tumor parenchyma. Integrating these modalities with SSIACI—where perfusion-guided catheter positioning already permits precise arterial targeting—could yield synergistic improvements in intratumoral drug concentration that neither approach achieves alone. Early clinical data from single-center cohorts and phase I trials have confirmed the technical feasibility and acceptable safety profile of SSIACI with osmotic BBB disruption in both adult and pediatric populations, and a recent systematic review and meta-analysis has reinforced the potential efficacy of selective and superselective intra-arterial infusion strategies across intracranial tumors. These findings collectively support the rationale for escalating toward larger, multi-institutional comparative trials.

Equally important is the integration of molecular and genomic profiling into endovascular treatment paradigms. The profound intratumoral heterogeneity of GBM—driven by glioma stem cells, MGMT-mediated resistance, mismatch repair deficiency, and adaptive immune evasion—demands that patient selection for IA therapies be guided by tumor-specific biomarkers rather than uniform protocols. Advances in artificial intelligence and multi-omics data integration offer the potential to predict treatment response, identify optimal therapeutic targets, and refine real-time procedural decision-making. Furthermore, the immunosuppressive tumor microenvironment, shaped by tumor-associated macrophages, myeloid-derived suppressor cells, and regulatory T cells, presents both a barrier and an opportunity: combining endovascular drug delivery with immunotherapeutic agents may help overcome local immune suppression while limiting the systemic toxicities that have hampered checkpoint inhibitor efficacy in GBM. Ultimately, the successful clinical translation of these multimodal strategies will require coordinated multidisciplinary collaboration among neurointerventionalists, neurosurgeons, neuro-oncologists, and translational scientists, alongside the development of standardized endpoints and adaptive trial designs capable of capturing the complexity of GBM biology.

## Figures and Tables

**Table 1:** Comparative Summary of BBB Disruption Strategies for Glioblastoma.

Therapeutic Agent	Class/Mechanism	Route(s) Investigated	Key Clinical Outcomes	Evidence Level/Approval Status	Major Limitations	References
Temozolomide	Alkylating agent; methylates DNA purines	Oral (standard); IA (investigational)	Stupp protocol: mOS 14.6 mo vs. 12.1 mo (RT alone); with TTF: mOS 20.9 mo; CNS levels ~30% of serum	Phase III RCTs; FDA-approved (standard of care)	Short half-life (1.8 h); MGMT-mediated resistance; dose-limiting thrombocytopenia/lymphopenia; active metabolite (MTIC) does not cross BBB	[52]
Bevacizumab	Anti-VEGF monoclonal antibody; inhibits tumor angiogenesis	IV (standard); IA via SSIACI (investigational)	Recurrent GBM: improved PFS but no OS benefit (CheckMate 143: mOS 9.8 mo nivo vs. 10.0 mo bev); newly diagnosed: PFS improvement (3–4 mo) but no OS benefit (AVAglio, RTOG 0825)	Phase III RCTs; FDA-approved for recurrent GBM	No OS benefit; selects for resistant, more aggressive phenotypes; promotes pseudovascular channels; hypertension, proteinuria, thromboembolic events	[52,53,54,55]
Lomustine (CCNU)	Nitrosourea alkylating agent; DNA crosslinking	Oral	Recurrent GBM: mOS ~8–9 mo; used as active comparator in multiple RCTs	Phase III (as comparator); NCCN preferred for recurrent GBM	Cumulative myelosuppression; pulmonary toxicity; limited single-agent efficacy	[42,56]
Carboplatin	Platinum-based; DNA crosslinking	IV; IA (with BBB disruption)	IA + BBBD: mOS 9.1 mo from treatment, 32.2 mo from diagnosis (Sherbrooke); implantable US + carboplatin: mOS 14.0 mo (recurrent)	Phase I/II; NCCN category 3 for recurrent GBM	Nephrotoxicity; myelosuppression; limited single-agent activity; IA requires specialized infrastructure	[42,57,58]
Nivolumab	Anti-PD-1 immune checkpoint inhibitor	IV	CheckMate 143: no OS benefit vs. bevacizumab (mOS 9.8 vs. 10.0 mo); CheckMate 498/548: no benefit in newly diagnosed GBM; NUTMEG: no benefit in elderly	Phase III RCTs; NOT approved for GBM	Immunosuppressive TME limits efficacy; BBB restricts immune cell infiltration; no validated predictive biomarkers for GBM	[59,60]
Tumor Treating Fields (TTF)	Alternating electric fields disrupt mitosis	Transducer arrays on scalp	EF-14: mOS 20.9 mo vs. 16.0 mo (TMZ alone); HR 0.63 (*p* = 0.001)	Phase III RCT; FDA-approved (newly diagnosed GBM)	Compliance-dependent (≥18 h/day); skin irritation; high cost; questions about mode of action and QOL impact	[52,53]
CAR-T Cell Therapy (EGFRvIII)	Engineered T cells targeting tumor-associated antigens	IV; intratumoral	Phase I: antigen loss and adaptive resistance after single dose; limited tumor infiltration	Phase I (investigational)	Antigen heterogeneity; limited tumor infiltration; manufacturing complexity (>$400,000/patient); 13–54 day production time	[52,61]
Dianhydrogalactitol (VAL-083)	Bifunctional alkylating agent; MGMT-independent DNA crosslinking (N7 guanine)	IV; IA (investigational)	Preclinical: overcomes TMZ resistance; clinical benefits modest in early trials	Phase I/II (investigational)	Limited clinical data; modest clinical benefit to date; requires further validation	[42]
EGFR-Targeted Therapies	Small molecules/antibodies targeting EGFR pathway	IV; IA (cetuximab via SSIACI)	No significant clinical efficacy in multiple Phase II/III trials despite strong biological rationale	Phase II/III (failed); cetuximab IA in Phase I	Tumor heterogeneity; adaptive resistance; pathway redundancy; poor CNS penetration of most agents	[52,56]
Regorafenib	Multi-kinase inhibitor (VEGFR, PDGFR, FGFR, KIT, RET, RAF)	Oral	REGOMA trial: mOS 7.4 mo vs. 5.6 mo (lomustine); HR 0.50 (*p* = 0.0009); one of few agents showing individual trial benefit	Phase II (single trial); NCCN does not list for GBM	Single-center Phase II; not confirmed in Phase III; hepatotoxicity; hand-foot skin reaction	[56]

## Data Availability

Data sharing is not applicable. No new data were created or analyzed in this study.

## References

[1] Price M., Ballard C., Benedetti J., Neff C., Cioffi G., Waite K.A., Kruchko C., Barnholtz-Sloan J.S., Ostrom Q.T. (2024). CBTRUS Statistical Report: Primary Brain and Other Central Nervous System Tumors Diagnosed in the United States in 2017–2021. Neuro Oncol..

[2] Schaff L.R., Mellinghoff I.K. (2023). Glioblastoma and Other Primary Brain Malignancies in Adults: A Review. JAMA.

[3] Ostrom Q.T., Price M., Neff C., Cioffi G., Waite K.A., Kruchko C., Barnholtz-Sloan J.S. (2023). CBTRUS Statistical Report: Primary Brain and Other Central Nervous System Tumors Diagnosed in the United States in 2016–2020. Neuro Oncol..

[4] Ostrom Q.T., Shoaf M.L., Cioffi G., Waite K., Kruchko C., Wen P.Y., Brat D.J., Barnholtz-Sloan J.S., Iorgulescu J.B. (2023). National-level overall survival patterns for molecularly-defined diffuse glioma types in the United States. Neuro Oncol..

[5] Ijzerman-Korevaar M., Snijders T.J., de Graeff A., Teunissen S.C.C.M., de Vos F.Y.F. (2018). Prevalence of symptoms in glioma patients throughout the disease trajectory: A systematic review. J. Neurooncol..

[6] Nabors L.B., Portnow J., Ahluwalia M., Baehring J., Brem H., Brem S., Butowski N., Campian J.L., Clark S.W., Fabiano A.J. (2020). Central Nervous System Cancers, Version 3.2020, NCCN Clinical Practice Guidelines in Oncology. J. Natl. Compr. Cancer Netw..

[7] Pöhlmann J., Weller M., Marcellusi A., Grabe-Heyne K., Krott-Coi L., Rabar S., Pollock R.F. (2024). High costs, low quality of life, reduced survival, and room for improving treatment: An analysis of burden and unmet needs in glioma. Front. Oncol..

[8] Norden A.D., Korytowsky B., You M., Le T.K., Dastani H., Bobiak S., Singh P. (2019). A Real-World Claims Analysis of Costs and Patterns of Care in Treated Patients with Glioblastoma Multiforme in the United States. J. Manag. Care Spec. Pharm..

[9] Stupp R., Mason W.P., van den Bent M.J., Weller M., Fisher B., Taphoorn M.J., Belanger K., Brandes A.A., Marosi C., Bogdahn U. (2005). Radiotherapy plus concomitant and adjuvant temozolomide for glioblastoma. N. Engl. J. Med..

[10] Jang H.J., Park J.W. (2025). Microenvironmental Drivers of Glioma Progression. Int. J. Mol. Sci..

[11] Khalafallah A.M., Huq S., Jimenez A.E., Serra R., Bettegowda C., Mukherjee D. (2021). “Zooming in” on Glioblastoma: Understanding Tumor Heterogeneity and its Clinical Implications in the Era of Single-Cell Ribonucleic Acid Sequencing. Neurosurgery.

[12] Tang J., Amin M.A., Campian J.L. (2025). Glioblastoma Stem Cells at the Nexus of Tumor Heterogeneity, Immune Evasion, and Therapeutic Resistance. Cells.

[13] Emir S.M., Karaoğlan B.S., Kaşmer R., Şirin H.B., Sarıyıldız B., Karakaş N. (2025). Hunting glioblastoma recurrence: Glioma stem cells as retrospective targets. Am. J. Physiol.-Cell Physiol..

[14] Bayona C., Ranđelović T., Ochoa I. (2026). Tumor microenvironment in glioblastoma: The central role of the hypoxic-necrotic core. Cancer Lett..

[15] Anwer M.S., Abdel-Rasol M.A., El-Sayed W.M. (2025). Emerging therapeutic strategies in glioblastsoma: Drug repurposing, mechanisms of resistance, precision medicine, and technological innovations. Clin. Exp. Med..

[16] Singh S., Dey D., Barik D., Mohapatra I., Kim S., Sharma M., Prasad S., Wang P., Singh A., Singh G. (2025). Glioblastoma at the crossroads: Current understanding and future therapeutic horizons. Signal Transduct. Target. Ther..

[17] Li Y., Chen Y., Cai K., Qin Y., Wang X., Zhang B., Shi L., He Z., Wang J., Long J. (2025). Interactions between glioblastoma and myeloid cells. Front. Cell Dev. Biol..

[18] Jackson C., Cherry C., Bom S., Dykema A.G., Wang R., Thompson E., Zhang M., Li R., Ji Z., Hou W. (2025). Distinct myeloid-derived suppressor cell populations in human glioblastoma. Science.

[19] Yalamarty S.S.K., Filipczak N., Li X., Subhan M.A., Parveen F., Ataide J.A., Rajmalani B.A., Torchilin V.P. (2023). Mechanisms of Resistance and Current Treatment Options for Glioblastoma Multiforme (GBM). Cancers.

[20] Mooney J., Bernstock J.D., Ilyas A., Ibrahim A., Yamashita D., Markert J.M., Nakano I. (2019). Current Approaches and Challenges in the Molecular Therapeutic Targeting of Glioblastoma. World Neurosurg..

[21] Lombardi G., Pambuku A., Bellu L., Farina M., Della Puppa A., Denaro L., Zagonel V. (2017). Effectiveness of antiangiogenic drugs in glioblastoma patients: A systematic review and meta-analysis of randomized clinical trials. Crit. Rev. Oncol. Hematol..

[22] Ghiaseddin A., Peters K.B. (2015). Use of bevacizumab in recurrent glioblastoma. CNS Oncol..

[23] Agarwala S.S., Kirkwood J.M. (2000). Temozolomide, a Novel Alkylating Agent with Activity in the Central Nervous System, May Improve the Treatment of Advanced Metastatic Melanoma. Oncologist.

[24] Reardon D.A., Brandes A.A., Omuro A., Mulholland P., Lim M., Wick A., Baehring J., Ahluwalia M.S., Roth P., Bähr O. (2020). Effect of Nivolumab vs Bevacizumab in Patients With Recurrent Glioblastoma: The CheckMate 143 Phase 3 Randomized Clinical Trial. JAMA Oncol..

[25] Steeg P.S. (2021). The blood–tumour barrier in cancer biology and therapy. Nat. Rev. Clin. Oncol..

[26] Ahmed M.H., Canney M., Carpentier A., Idbaih A. (2023). Overcoming the blood brain barrier in glioblastoma: Status and future perspective. Rev. Neurol..

[27] Pu J., Yuan K., Tao J., Qin Y., Li Y., Fu J., Li Z., Zhou H., Tang Z., Li L. (2025). Glioblastoma multiforme: An updated overview of temozolomide resistance mechanisms and strategies to overcome resistance. Discov. Oncol..

[28] Branch P., Aquilina G., Bignami M., Karran P. (1993). Defective mismatch binding and a mutator phenotype in cells tolerant to DNA damage. Nature.

[29] Jiménez-Alcázar M., Curiel-García Á., Nogales P., Perales-Patón J., Schuhmacher A.J., Galán-Ganga M., Zhu L., Lowe S.W., Al-Shahrour F., Squatrito M. (2021). Dianhydrogalactitol Overcomes Multiple Temozolomide Resistance Mechanisms in Glioblastoma. Mol. Cancer Ther..

[30] Stupp R., Taillibert S., Kanner A., Read W., Steinberg D., Lhermitte B., Toms S., Idbaih A., Ahluwalia M.S., Fink K. (2017). Effect of Tumor-Treating Fields Plus Maintenance Temozolomide vs Maintenance Temozolomide Alone on Survival in Patients With Glioblastoma: A Randomized Clinical Trial. JAMA.

[31] Tan A.C., Ashley D.M., López G.Y., Malinzak M., Friedman H.S., Khasraw M. (2020). Management of glioblastoma: State of the art and future directions. CA Cancer J. Clin..

[32] O’Rourke D.M., Nasrallah M.P., Desai A., Melenhorst J.J., Mansfield K., Morrissette J.J.D., Martinez-Lage M., Brem S., Maloney E., Shen A. (2017). A single dose of peripherally infused EGFRvIII-directed CAR T cells mediates antigen loss and induces adaptive resistance in patients with recurrent glioblastoma. Sci. Transl. Med..

[33] Beck A., Goetsch L., Dumontet C., Corvaïa N. (2017). Strategies and challenges for the next generation of antibody–drug conjugates. Nat. Rev. Drug Discov..

[34] Gonzalez Castro L.N., Santagata S. (2026). Antibody-drug conjugates for primary brain tumors. Front. Oncol..

[35] Shi J., Kantoff P.W., Wooster R., Farokhzad O.C. (2017). Cancer nanomedicine: Progress, challenges and opportunities. Nat. Rev. Cancer.

[36] Sarfraz Z., Ranjan T., Mustafayev F.N.A., Jaramillo M., Odia Y., Venur V.A., Ahluwalia M.S. (2025). Emerging therapies for glioblastoma. J. Neurooncol..

[37] Mohile N.A., Messersmith H., Gatson N.T., Hottinger A.F., Lassman A., Morton J., Ney D., Nghiemphu P.L., Olar A., Olson J. (2022). Therapy for Diffuse Astrocytic and Oligodendroglial Tumors in Adults: ASCO-SNO Guideline. J. Clin. Oncol..

[38] Daneman R., Prat A. (2015). The blood-brain barrier. Cold Spring Harb. Perspect. Biol..

[39] Neuwelt E., Abbott N.J., Abrey L., Banks W.A., Blakley B., Davis T., Engelhardt B., Grammas P., Nedergaard M., Nutt J. (2008). Strategies to advance translational research into brain barriers. Lancet Neurol..

[40] Hynynen K., McDannold N., Vykhodtseva N., Jolesz F.A. (2001). Noninvasive MR imaging-guided focal opening of the blood-brain barrier in rabbits. Radiology.

[41] Lee E.J., Fomenko A., Lozano A.M. (2019). Magnetic Resonance-Guided Focused Ultrasound: Current Status and Future Perspectives in Thermal Ablation and Blood-Brain Barrier Opening. J. Korean Neurosurg. Soc..

[42] Woodworth G.F., Anastasiadis P., Ozair A., Chabros J., Bettegowda C., Chen C., Gerstl J.V.E., Douville C., Mekary R.A., Smith T.R. (2025). Microbubble-enhanced transcranial focused ultrasound with temozolomide for patients with high-grade glioma (BT008NA): A multicentre, open-label, phase 1/2 trial. Lancet Oncol..

[43] Sonabend A.M., Gould A., Amidei C., Ward R., Schmidt K.A., Zhang D.Y., Gomez C., Bebawy J.F., Liu B.P., Bouchoux G. (2023). Repeated blood-brain barrier opening with an implantable ultrasound device for delivery of albumin-bound paclitaxel in patients with recurrent glioblastoma: A phase 1 trial. Lancet Oncol..

[44] Chen S.R., Chen M.M., Ene C., Lang F.F., Kan P. (2022). Perfusion-guided endovascular super-selective intra-arterial infusion for treatment of malignant brain tumors. J. NeuroInterv. Surg..

[45] Young C.C., Narsinh K.H., Chen S.R., Ansari S.A., Hetts S.W., Lang F.F., Wintermark M., Kan P.T. (2026). A Report from the Inaugural Society of NeuroInterventional Surgery Neurointerventional Oncology Summit. AJNR Am. J. Neuroradiol..

[46] Paolani G., Minosse S., Strolin S., Santoro M., Pucci N., Di Giuliano F., Garaci F., Oddo L., Toumia Y., Guida E. (2025). Intra-Arterial Super-Selective Delivery of Yttrium-90 for the Treatment of Recurrent Glioblastoma: In Silico Proof of Concept with Feasibility and Safety Analysis. Pharmaceutics.

[47] Saraiva C., Praça C., Ferreira R., Santos T., Ferreira L., Bernardino L. (2016). Nanoparticle-mediated brain drug delivery: Overcoming blood-brain barrier to treat neurodegenerative diseases. J. Control. Release.

[48] Tosi G., Duskey J.T., Kreuter J. (2020). Nanoparticles as carriers for drug delivery of macromolecules across the blood-brain barrier. Expert Opin. Drug Deliv..

[49] Georgieva J.V., Hoekstra D., Zuhorn I.S. (2014). Smuggling Drugs into the Brain: An Overview of Ligands Targeting Transcytosis for Drug Delivery across the Blood-Brain Barrier. Pharmaceutics.

[50] Pardridge W.M. (2012). Drug transport across the blood-brain barrier. J. Cereb. Blood Flow Metab..

[51] Pardridge W.M. (2007). Blood-brain barrier delivery. Drug Discov. Today.

[52] Ferreira C., Ferreira M.Y., Cardoso L.J.C., Scarramal J.P.L., Nogueira A., Wong T., Bokil S., Singh F., Massimo S., Albers O. (2026). Safety and efficacy of selective and superselective intra-arterial cerebral infusion with blood-brain barrier disruption for glioma: A systematic review and meta-analysis. J. NeuroInterv. Surg..

[53] Rahmanipour E., Ghorbani M., Demir E., Gupta K., Sani G.G., Patillo C., Pahlevani M., Baloi D., Samieirad S., Daskareh M. (2026). Efficacy of intra-arterial chemotherapy across intracranial tumors: A systematic review and meta-analysis. Crit. Rev. Oncol. Hematol..

[54] Gahide G., Vendrell J.F., Massicotte-Tisluck K., Caux S., Deschamps S., Noël-Lamy M., Belzile F., Roy L.O., Fortin D. (2025). Safety of Cerebral Intra-Arterial Chemotherapy for the Treatment of Malignant Brain Tumours. J. Clin. Med..

[55] Dufault M.E., Akhavan-Sigari A., Pachón-Londoño M.J., Olson V.A., Turcotte E.L., Beniwal A.S., Carlson B.H., Moussalem C.K., Giotta Lucifero A., Bathini A.R. (2026). From barrier to bridge: A scoping review on the methods, clinical efficacy, and safety of blood-brain barrier disruption in treating high-grade glioma. J. Neurooncol..

[56] Alrashidi M., Ferro F., Almohammadi A., Alyoubi N.H., Alsarheed G.S., Joseph J., Banerjee S. (2026). Efficacy and safety of low- and high-intensity focused ultrasound in glioblastoma: A systematic review of preclinical and clinical studies. Br. J. Cancer.

[57] Carpentier A., Stupp R., Sonabend A.M., Dufour H., Chinot O., Mathon B., Ducray F., Guyotat J., Baize N., Menei P. (2024). Repeated blood-brain barrier opening with a nine-emitter implantable ultrasound device in combination with carboplatin in recurrent glioblastoma: A phase I/II clinical trial. Nat. Commun..

[58] Vargas L.O., Himic V., Otaner F., Abikenari M., Chandar J., Govindarajan V., Kreatsoulas D., Jahangiri A., Komotar R.J., Ivan M.E. (2025). Modulating the glioma microenvironment with laser interstitial thermal therapy: Mechanisms and therapeutic implications. J. Neurooncol..

[59] Cleary R.T., Fu Y., Giles D., Yuan J., Moniz Garcia D.P., Palmer D., Han R.H., Woodiwiss T., Yang A.B., Mathios D. (2026). Laser Interstitial Thermal Therapy Enhances Bidirectional Blood-Brain Barrier Permeability in Glioblastoma. Neuro Oncol..

[60] Piper K., Kumar J.I., Domino J., Tuchek C., Vogelbaum M.A. (2024). Consensus review on strategies to improve delivery across the blood-brain barrier including focused ultrasound. Neuro Oncol..

[61] Bérard C., Desgranges S., Dumas N., Novell A., Selingue E., Hamimed M., Chevallier O.P., Truillet C., Larrat B., Taulier N. (2026). Combined Docetaxel-Loaded Perfluorocarbon Nanodroplets with Ultrasound-Mediated Blood-Brain Barrier Disruption for Effective Glioblastoma Treatment in Mice Model. Int. J. Nanomed..

[62] Huang R., Boltze J., Li S. (2020). Strategies for Improved Intra-arterial Treatments Targeting Brain Tumors: A Systematic Review. Front. Oncol..

[63] Joshi S., Ellis J.A., Ornstein E., Bruce J.N. (2015). Intraarterial drug delivery for glioblastoma mutiforme: Will the phoenix rise again?. J. Neurooncol..

[64] Schuster J.M., Friedman H.S., Archer G.E., Fuchs H.E., McLendon R.E., Colvin O.M., Bigner D.D. (1993). Intraarterial therapy of human glioma xenografts in athymic rats using 4-hydroperoxycyclophosphamide. Cancer Res..

[65] Kurpad S.N., Friedman H.S., Archer G.E., McLendon R.E., Petros W.M., Fuchs H.E., Guaspari A., Bigner D.D. (1995). Intraarterial administration of melphalan for treatment of intracranial human glioma xenografts in athymic rats. Cancer Res..

[66] Qureshi A.I. (2004). Endovascular treatment of cerebrovascular diseases and intracranial neoplasms. Lancet.

[67] Srinivasan V.M., Lang F.F., Chen S.R., Chen M.M., Gumin J., Johnson J., Burkhardt J.K., Kan P. (2020). Advances in endovascular neuro-oncology: Endovascular selective intra-arterial (ESIA) infusion of targeted biologic therapy for brain tumors. J. NeuroInterv. Surg..

[68] Kappel A.D., Jha R., Guggilapu S., Smith W.J., Feroze A.H., Dmytriw A.A., Vicenty-Padilla J., Alcedo Guardia R.E., Gessler F.A., Patel N.J. (2024). Endovascular Applications for the Management of High-Grade Gliomas in the Modern Era. Cancers.

[69] Janowski M., Walczak P., Pearl M.S. (2016). Predicting and optimizing the territory of blood-brain barrier opening by superselective intra-arterial cerebral infusion under dynamic susceptibility contrast MRI guidance. J. Cereb. Blood Flow Metab..

[70] Latulippe J., Roy L.O., Gobeil F., Fortin D. (2025). Optimization of Intra-Arterial Administration of Chemotherapeutic Agents for Glioblastoma in the F98-Fischer Glioma-Bearing Rat Model. Biomolecules.

[71] Qureshi A.I., Suri M.F., Khan J., Sharma M., Olson K., Guterman L.R., Hopkins L.N. (2001). Superselective intra-arterial carboplatin for treatment of intracranial neoplasms: Experience in 100 procedures. J. Neurooncol..

[72] Fortin D., Desjardins A., Benko A., Niyonsega T., Boudrias M. (2005). Enhanced chemotherapy delivery by intraarterial infusion and blood-brain barrier disruption in malignant brain tumors: The Sherbrooke experience. Cancer.

[73] Angelov L., Doolittle N.D., Kraemer D.F., Siegal T., Barnett G.H., Peereboom D.M., Stevens G., McGregor J., Jahnke K., Lacy C.A. (2009). Blood-brain barrier disruption and intra-arterial methotrexate-based therapy for newly diagnosed primary CNS lymphoma: A multi-institutional experience. J. Clin. Oncol..

[74] Willinsky R.A., Taylor S.M., TerBrugge K., Farb R.I., Tomlinson G., Montanera W. (2003). Neurologic complications of cerebral angiography: Prospective analysis of 2,899 procedures and review of the literature. Radiology.

[75] Lewandowski R.J., Geschwind J.F., Liapi E., Salem R. (2011). Transcatheter intraarterial therapies: Rationale and overview. Radiology.

[76] Shin B.J., Burkhardt J.K., Riina H.A., Boockvar J.A. (2012). Superselective intra-arterial cerebral infusion of novel agents after blood-brain disruption for the treatment of recurrent glioblastoma multiforme: A technical case series. Neurosurg. Clin. N. Am..

[77] Doolittle N.D., Miner M.E., Hall W.A., Siegal T., Jerome E., Osztie E., McAllister L.D., Bubalo J.S., Kraemer D.F., Fortin D. (2000). Safety and efficacy of a multicenter study using intraarterial chemotherapy in conjunction with osmotic opening of the blood-brain barrier for the treatment of patients with malignant brain tumors. Cancer.

[78] Burkhardt J.K., Shin B.J., Schlaff C.D., Riina H., Boockvar J.A. (2011). Cost analysis of intra-arterial versus intra-venous delivery of bevacizumab for the treatment of recurrent glioblastoma multiforme. J. Exp. Ther. Oncol..

[79] Pinkiewicz M., Pinkiewicz M., Walecki J., Zawadzki M. (2022). A systematic review on intra-arterial cerebral infusions of chemotherapeutics in the treatment of glioblastoma multiforme: The state-of-the-art. Front. Oncol..

[80] Wu D., Chen Q., Chen X., Han F., Chen Z., Wang Y. (2023). The blood–brain barrier: Structure, regulation and drug delivery. Signal Transduct. Target. Ther..

[81] Burkhardt J.K., Riina H., Shin B.J., Christos P., Kesavabhotla K., Hofstetter C.P., Tsiouris A.J., Boockvar J.A. (2012). Intra-arterial delivery of bevacizumab after blood-brain barrier disruption for the treatment of recurrent glioblastoma: Progression-free survival and overall survival. World Neurosurg..

[82] Riina H.A., Fraser J.F., Fralin S., Knopman J., Scheff R.J., Boockvar J.A. (2009). Superselective intraarterial cerebral infusion of bevacizumab: A revival of interventional neuro-oncology for malignant glioma. J. Exp. Ther. Oncol..

[83] Duerinck J., Tuyaerts S., Movahedi K., Neyns B. (2023). Overcoming the immune suppressive nature of glioblastoma by leveraging the surgical intervention—current status and future perspectives. Front. Immunol..

[84] McCrea H.J., Ivanidze J., O’Connor A., Hersh E.H., Boockvar J.A., Gobin Y.P., Knopman J., Greenfield J.P. (2021). Intraarterial delivery of bevacizumab and cetuximab utilizing blood-brain barrier disruption in children with high-grade glioma and diffuse intrinsic pontine glioma: Results of a phase I trial. J. Neurosurg. Pediatr..

[85] Ferreira C., Ferreira M.Y., Singh F., Wong T., Bokil S., Massimo S., Cavallaro J., Albers O., D’Amico R., Langer D. (2026). Superselective intra-arterial cerebral infusion of chemotherapeutics after osmotic blood-brain barrier disruption in newly diagnosed or recurrent glioblastoma: Technical insights and clinical outcomes from a single-center experience. J. NeuroInterv. Surg..

[86] Faltings L., Kulason K.O., Patel N.V., Wong T., Fralin S., Li M., Schneider J.R., Filippi C.G., Langer D.J., Ortiz R. (2019). Rechallenging Recurrent Glioblastoma with Intra-Arterial Bevacizumab with Blood Brain-Barrier Disruption Results in Radiographic Response. World Neurosurg..

[87] Shapiro W.R., Green S.B., Burger P.C., Selker R.G., VanGilder J.C., Robertson J.T., Mealey J., Ransohff J., Mahaley M.S. (1992). A randomized comparison of intra-arterial versus intravenous BCNU, with or without intravenous 5-fluorouracil, for newly diagnosed patients with malignant glioma. J. Neurosurg..

[88] Chen W., Wu Q., Mo L., Nassi M. (2013). Intra-arterial chemotherapy is not superior to intravenous chemotherapy for malignant gliomas: A systematic review and meta-analysis. Eur. Neurol..

[89] Theodotou C., Shah A.H., Hayes S., Bregy A., Johnson J.N., Aziz-Sultan M.A., Komotar R.J. (2014). The role of intra-arterial chemotherapy as an adjuvant treatment for glioblastoma. Br. J. Neurosurg..

[90] Rechberger J.S., Thiele F., Daniels D.J. (2021). Status Quo and Trends of Intra-Arterial Therapy for Brain Tumors: A Bibliometric and Clinical Trials Analysis. Pharmaceutics.

[91] Patel N.V., Wong T., Fralin S.R., Li M., McKeown A., Gruber D., D’Amico R.S., Patsalides A., Tsiouris A., Stefanov D.G. (2021). Repeated superselective intraarterial bevacizumab after blood brain barrier disruption for newly diagnosed glioblastoma: A phase I/II clinical trial. J. Neurooncol..

[92] Kirby S., Brothers M., Irish W., Florell R., Macdonald D., Schold C., Cairncross G. (1995). Evaluating glioma therapies: Modeling treatments and predicting outcomes. J. Natl. Cancer Inst..

[93] Sainatham C., Yadav D., Dilli Babu A., Tallapalli J.R., Kanagala S.G., Filippov E., Murillo Chavez F., Ahmed N., Lutfi F. (2024). The current socioeconomic and regulatory landscape of immune effector cell therapies. Front. Med..

[94] Brudno J.N., Maus M.V., Hinrichs C.S. (2024). CAR T Cells and T-Cell Therapies for Cancer: A Translational Science Review. JAMA.

[95] Ma P., Li Y., Gu Y., Zeng H., Xiang H., Cao Z., Han Y., Cui Y., Liu H. (2025). Advances and challenges in novel drug delivery systems for glioma therapy. Front. Pharmacol..

[96] Kim Y., Armstrong T.S., Gilbert M.R., Celiku O. (2023). A critical analysis of neuro-oncology clinical trials. Neuro Oncol..

[97] Shah H.A., Mishra A., Gouzoulis M.J., Ben-Shalom N., D’Amico R.S. (2022). Analysis of factors leading to early termination in glioblastoma-related clinical trials. J. Neurooncol..

[98] Rotte A. (2025). Development of Cell and Gene Therapies for Clinical Use in the US and EU: Summary of Regulatory Guidelines. Curr. Gene Ther..

[99] Walton C.M., Bell M., O’Neil R., Sahin O., Choi B.D., Fecci P.E., Strickland B.A. (2025). Chimeric antigen receptor (CAR) T-cell therapy for glioblastoma (GBM): Current clinical insights, challenges, and future directions. J. Immunother. Cancer.

[100] Rahman R., Polley M.C., Alder L., Brastianos P.K., Anders C.K., Tawbi H.A., Mehta M., Wen P.Y., Geyer S., de Groot J. (2023). Current drug development and trial designs in neuro-oncology: Report from the first American Society of Clinical Oncology and Society for Neuro-Oncology Clinical Trials Conference. Lancet Oncol..

[101] Agrawal S., Vaidya S., Patel J., Jirvankar P., Gurjar P. (2025). Challenges and Pathways in Regulating Next-Gen Biological Therapies. Curr. Pharm. Biotechnol..

[102] Wisdom A.J., Temple H., Cui Y., Del Vecchio C.T., Kim T., White F.M., Floyd S.R., Spranger S. (2026). Mechanisms, Microenvironments, and Models: Understanding Therapeutic Resistance in Glioblastoma. Int. J. Radiat. Oncol. Biol. Phys..

[103] Omran N.E., Zenati R.A., Bou Malhab L.J., Bustanji Y., Alzoubi K.H., Harati R., Semreen M.H. (2026). Understanding resistance in glioblastoma: Insights into personalized and targeted therapeutic strategies. Neuroscience.

[104] Beylerli O., Gareev I., Musaev E., Ilyasova T., Roumiantsev S., Chekhonin V. (2026). Angiogenesis and Resistance Mechanisms in Glioblastoma: Targeting Alternative Vascularization Pathways to Overcome Therapy Resistance. Curr. Pharm. Des..

[105] Li Y., Du Y., Guan F., Li X., Cui S., Gao W., Long X., Wu M. (2026). Dissecting the aberrant vasculature of glioblastoma: Mechanisms and therapeutic targets. Cancer Metastasis Rev..

[106] Salazar-Saura I., Pinilla-Sala M., Megías J., Navarro L., Roselló-Sastre E., San-Miguel T. (2024). Pericytes in Glioblastoma: Hidden Regulators of Tumor Vasculature and Therapy Resistance. Cancers.

[107] Schupper A.J., Hadjipanayis C.G. (2023). Novel approaches to targeting gliomas at the leading/cutting edge. J. Neurosurg..

[108] Pichol-Thievend C., Anezo O., Pettiwala A.M., Bourmeau G., Montagne R., Lyne A.M., Guichet P.O., Deshors P., Ballestín A., Blanchard B. (2024). VC-resist glioblastoma cell state: Vessel co-option as a key driver of chemoradiation resistance. Nat. Commun..

[109] Lang F., Liu Y., Chou F.J., Yang C. (2021). Genotoxic therapy and resistance mechanism in gliomas. Pharmacol. Ther..

[110] Abuhassan Q., Al-Ameer H.J., Ganesan S., Nayak P.P., Kumar V.R., Sharma V., Singh Chauhan A., Goyibova N. (2026). Interactions and communications in the glioblastoma microenvironment: Potential targets for chemo-/radiotherapy. Strahlenther. Onkol..

[111] Lim S.H., Yee G.T., Khang D. (2024). Nanoparticle-Based Combinational Strategies for Overcoming the Blood-Brain Barrier and Blood-Tumor Barrier. Int. J. Nanomed..

[112] Brambila C., Sahebi Vaighan N., Youssef I., Chaudhary J., Anwar A., Bachoo R., Patel T., Chopra R., Corbin I.R. (2026). Enhanced delivery of low-density lipoprotein-based nanoparticles to the mouse glioblastoma using focused ultrasound as a novel therapy. Biomaterials.

[113] Virtanen P.S., Ortiz K.J., Patel A., Blocher W.A., Richardson A.M. (2024). Blood-Brain Barrier Disruption for the Treatment of Primary Brain Tumors: Advances in the Past Half-Decade. Curr. Oncol. Rep..

[114] Weller J., Potthoff A.L., Zeyen T., Schaub C., Duffy C., Schneider M., Herrlinger U. (2024). Current status of precision oncology in adult glioblastoma. Mol. Oncol..

[115] Alnahhas I. (2024). Molecular Testing in Gliomas: What is Necessary in Routine Clinical Practice?. Curr. Oncol. Rep..

[116] Kakde S.P., Kakde M.P., Arora N., Kakade S.P. (2026). AI–assisted survival prediction in glioma: Meta-analysis of prospective and controlled studies. J. Clin. Oncol..

[117] Cepeda S., Hernando-Pérez E., Pérez-Riesgo E., Rodríguez-Valle I., Esteban-Sinovas O., Arrese I., Lucero-Salaverry M.M., Zamora T., Torres-Nieto M., Luppino L.T. (2026). Prospective biopsy-controlled validation of an AI model for predicting glioblastoma infiltration: Results from the SupraGlio trial. Neuro Oncol..

[118] Abuhassan Q., Al-Ameer H.J., Patel P.N., Jyothi S.R., Singh G., Maharana L., Arora V., Khalikova N. (2026). Integrating multi-omics data and artificial intelligence for personalized medicine in glioblastoma. Clin. Chim. Acta.

[119] Luo J., Pan M., Mo K., Mao Y., Zou D. (2023). Emerging role of artificial intelligence in diagnosis, classification and clinical management of glioma. Semin. Cancer Biol..

[120] Khalighi S., Reddy K., Midya A., Pandav K.B., Madabhushi A., Abedalthagafi M. (2024). Artificial intelligence in neuro-oncology: Advances and challenges in brain tumor diagnosis, prognosis, and precision treatment. npj Precis. Oncol..

[121] Awuah W.A., Ben-Jaafar A., Roy S., Nkrumah-Boateng P.A., Tan J.K., Abdul-Rahman T., Atallah O. (2025). Predicting survival in malignant glioma using artificial intelligence. Eur. J. Med. Res..

